# Multichannel and Wide-Angle SAR Imaging Based on Compressed Sensing

**DOI:** 10.3390/s17020295

**Published:** 2017-02-05

**Authors:** Chao Sun, Baoping Wang, Yang Fang, Zuxun Song, Shuzhen Wang

**Affiliations:** 1 School of Electronics and Information, Northwestern Polytechnical University, Xi’an 710129, China; fangyang@mail.nwpu.edu.cn (Y.F.); zxsong@nwpu.edu.cn (Z.S.); 2 Science and Technology on UAV Laboratory, Northwestern Polytechnical University, Xi’an 710065, China; 3 School of Computer Science and Technology, Xidian University, Xi’an 710071, China; shuzhenwang@xidian.edu.cn

**Keywords:** synthetic aperture radar, multichannel, wide-angle, compressed sensing, joint sparse recovery

## Abstract

The multichannel or wide-angle imaging performance of synthetic aperture radar (SAR) can be improved by applying the compressed sensing (CS) theory to each channel or sub-aperture image formation independently. However, this not only neglects the complementary information between signals of each channel or sub-aperture, but also may lead to failure in guaranteeing the consistency of the position of a scatterer in different channel or sub-aperture images which will make the extraction of some scattering information become difficult. By exploiting the joint sparsity of the signal ensemble, this paper proposes a novel CS-based method for joint sparse recovery of all channel or sub-aperture images. Solving the joint sparse recovery problem with a modified orthogonal matching pursuit algorithm, the recovery precision of scatterers is effectively improved and the scattering information is also preserved during the image formation process. Finally, the simulation and real data is used for verifying the effectiveness of the proposed method. Compared with single channel or sub-aperture independent CS processing, the proposed method can not only obtain better imaging performance with fewer measurements, but also preserve more valuable scattering information for target recognition.

## 1. Introduction

As an active microwave remote sensing imaging tool, synthetic aperture radar (SAR) has the unique capability of obtaining abundant electromagnetic information from ground objects throughout the day and night under all-weather circumstances. As such, SAR imaging has been widely utilized in many military and civilian applications [1,2]. The development of multichannel and wide-angle SAR imaging greatly broadens the application field of SAR. Interferometric SAR (InSAR) and polarimetric SAR (PolSAR) are two representative forms of multichannel SAR. InSAR can obtain three-dimensional (3-D) topographic mapping data and the ground surface deformation monitoring from the phase difference of the corresponding pixels of two SAR images received by two spatially separated antennas or locations [3,4]. PolSAR focuses on transmitting and receiving polarized radar waves to characterize the observed target. For the sake of polarimetric scattering characteristic analysis, the polarimetric scattering matrix is firstly extracted from SAR images generated by different polarization channels [5]. In a wide-angle SAR imaging scenario, the isotropic point scattering assumption employed by conventional imaging does not usually hold, leading to inaccuracies in the relative reflectivities of scatterers with different levels of anisotropy. Yet, such aspect dependence can itself be an important feature for scene interpretation and target recognition. The composite image formation is usually used for producing a reflectivity estimate for each spatial location and some information on aspect dependence. The idea is to form sub-aperture images from narrower-angle subsets of the data, and then form a composite image through a nonlinear combination of these sub-aperture images [6].

In SAR applications, higher image resolutions are usually essential to determine finer parts of the target for better identification of the target. Conventionally, a finer azimuth resolution of an SAR image requires a longer coherent accumulation time and a finer range resolution is achieved by increasing the system bandwidth. Limited by the Nyquist sampling theorem, the SAR system faces a number of challenges, such as very high sampling rate, a huge amount of sampled data and difficulty of real-time processing. In particular, for InSAR and PolSAR imaging systems, the data volume may increase by a factor of 2 and 4, respectively, which will further increase the challenges as mentioned above.

The recently introduced theory of compressed sensing (CS) provides a new sampling paradigm that is able to reconstruct the sparse or compressible signals exactly from limited measurements by solving an optimization problem [7,8]. In radar imaging, according to CS theory, if the scene is sparse or compressible, it is sufficient to use only a few samples to reconstruct a high-resolution SAR image. Fortunately, many radar scenes are spatially sparse or can be sparsely represented by time-frequency transform [9], wavelet transform [10] or adaptive sparse transform [11], etc., which intrinsically provides a foundation to apply CS in generating high-resolution SAR imagery. In [12], CS is applied to range compression to eliminate the need for the pulse compression matched filter with reduced samples. For the strip-map SAR, Alonso et al. successfully reconstructs the SAR images with reduced pulses in azimuth after range compression [13]. As these methods apply CS to either range or azimuth compression, they are regarded as one-dimensional (1-D) CS-based SAR imaging methods. Two-dimensional (2-D) CS-based SAR imaging methods and some accelerated algorithms are proposed in [14,15,16,17,18]. In order to improve the robustness of the CS-based methods, some CS-based SAR imaging methods take phase errors [19,20] or off-grid problem [21,22] into account during the imaging process. Besides, CS-based methods for tomography SAR imaging have been successfully applied in recent years, obtaining high-resolution 3-D images [23,24,25]. Exploiting the statistics of the target’s distribution, structured sparse recovery methods are proposed to further improve performance in high-resolution SAR imaging [10,26,27,28]. All of the above mentioned works strongly demonstrated that some advantages of CS-based SAR do exist as compared with traditional SAR imaging methodologies, say, relaxation of required measurements, reduction of sidelobes, and a further suppression of noise.

However, all those works are based on single channel or narrow-angle SAR systems. For multichannel SAR systems, such as InSAR and PolSAR, we can directly process each channel independently using those exiting CS-based SAR imaging methods, and then extract height or polarization information from multichannel SAR images. Similarly, for wide-angle SAR imaging, we can directly process each sub-aperture independently using those exiting CS-based SAR imaging methods, and then extract the aspect scattering information from these sub-aperture SAR images. It is a fact that the dominant scatterers of the target locate on the same positions in different channel or sub-aperture images, namely, sharing the same sparsity support. Single channel or sub-aperture CS independent processing neglects this fact, and thus cannot guarantee the consistency of the number and the positions of scatterers in different channel or sub-aperture images, leading to difficulties in the extraction of some scattering information [29,30,31]. Besides, due to the imaging scene being the same in each channel or sub-aperture, there exists abundant complementary information between signals of each channel or sub-aperture, but independent CS processing cannot exploit the complementary information. If the complementary information is used, namely joint processing of all channels or sub-apertures, high-quality SAR images can be reconstructed with fewer measurements and the noise suppression ability can also be improved, compared with independent CS processing.

To overcome the existing problems of independent CS processing when applied to multichannel or wide-angle SAR imaging, in this paper, we derive a class of signal models for multichannel or wide-angle SAR imaging, and propose a novel CS-based method for joint high-resolution imaging and scattering information preservation. Exploiting the joint sparsity of the signal ensemble, multichannel or wide-angle SAR imaging is handled as a multichannel joint sparse recovery problem, which is efficiently solved by modified orthogonal matching pursuit algorithm. SAR images of all channels or sub-apertures are reconstructed simultaneously. Meanwhile, it guarantees that the number and the positions of scatterers in each channel or sub-aperture are consistent. Due to the usage of complementary information, the proposed method can reconstruct high-resolution focused SAR images with fewer measurements and under lower SNR, compared with independent CS processing. At last, the experimental data processing results are shown to demonstrate the validity of the proposed method.

The rest of this paper is organized as follows: in Section 2, the observation model of multichannel or wide-angle SAR system is described. In Section 3, independent CS and joint CS processing methods are presented, respectively. Section 4 provides the experimental results to verify the proposed method. Finally, in Section 5, some conclusions are summarized, and the future work outlook is given as well.

## 2. Signal Model

In SAR systems, one of the most widely used transmitted signals is the chirp signal as follows:

(1) $$s(t_{r})=\mathrm{rect}\left(\frac{t_{r}}{T_{P}}\right)\cdot \exp\left[j2\pi \left(f_{c}t_{r}+\frac{\gamma }{2}t_{r}^{2}\right)\right]$$

where *t_r_* denotes the fast time (range time), *f_c_* is the carrier frequency, *γ* is the chirp rate, *T_p_* denotes the pulse width, and rect(*t_r_*/*T_p_*) stands for the unit rectangular function. There are in total *L* channels in the multichannel SAR system, and the received signal of the *l*-th channel can be expressed as:

(2) $$s_{l}(t_{r},t_{a})=\iint_{D}g_{l}(x,y)\exp\left\{j\pi \gamma {\left[t_{r}-\frac{2R_{l}(t_{a},x,y)}{c}\right]}^{2}\right\}\exp\left[-j\frac{4\pi f_{c}}{c}R_{l}(t_{a},x,y)\right]dxdy$$

where *c* is the speed of light, *t_a_* is the slow time (azimuth time), *D* is the region to be imaged, (*x,y*) is the coordinates of the target, *g_l_*(*x,y*) is the backward scattering amplitude of the target in the *l*-th channel, and *R_l_*(*t_a_*,*x,y*) is the range from the target to the antenna of *l*-th channel at time *t_a_*.

The scene to be imaged is firstly discretized to a 2-D matrix. Then, to denote the radar data in a matrix multiplication form, the 2-D reflectivity coefficient matrix *g_l_*(*x,y*) is reshaped to a 1-D vector:

(3) $$g_{l}={\left[g_{l}(1,1),\cdots ,g_{l}(P,1),g_{l}(1,2),\cdots ,g_{l}(P,2),\cdots ,g_{l}(1,Q),\cdots ,g_{l}(P,Q)\right]}^{T}$$

where [·]*^T^* denotes a vector or matrix transpose, $g_{l}$ is a PQ×1 vector, *PQ* is the total number of discrete spatial locations in the scene. *P* and *Q* are the number of locations along the *x*-axis and *y*-axis.

**Figure 1:**
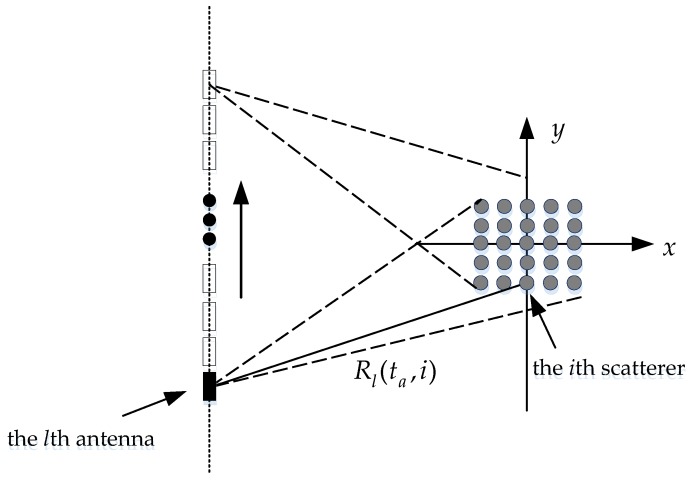
Geometry model for multichannel SAR imaging.

Based on Equation (2), the discrete normalized spatial expression of the radar data is given by:

(4) $$s_{l}(t_{r},t_{a})=\sum_{i=1}^{PQ}g_{l}(i)\exp\left\{j\pi \gamma {\left[t_{r}-\frac{2R_{l}(t_{a},i)}{c}\right]}^{2}\right\}\exp\left[-j\frac{4\pi f_{c}}{c}R_{l}(t_{a},i)\right]$$

where *g_l_*(*i*) is the backscattering amplitude of the *i*-th scatterer (i.e., the *i*-th element in $g_{l}$ ), and *R_l_*(*t_a_*,*i*) is the distance from the *i*-th scatterer to the antenna of the *l*-th channel at azimuth time *t_a_*. The imaging geometry is shown in Figure 1.

In practice, the range and azimuth times are also discrete due to the sampling process. The 2-D discrete SAR time signal can be expressed as:

(5) $$\begin{array}{l} s_{l}(t_{r,n},t_{a,m})=\sum_{i=1}^{PQ}g_{l}(i)\exp\left[-j\frac{4\pi f_{c}}{c}R(t_{a,m},i)\right]\exp\left\{j\pi \gamma {\left[t_{r,n}-\frac{2R(t_{a,m},i)}{c}\right]}^{2}\right\}\\ n=0,1,\cdots ,N-1;m=0,1,\cdots ,M-1 \end{array}$$

where *M* is the number of azimuth samples and *N* is the number of samples for each pulse.

Considering the noise in the signal model, Equation (5) can be expressed in a matrix form as:

(6) $$s_{l}=A_{l}g_{l}+e_{l}$$

where $s_{l}$ is an MN×1 vector, $A_{l}$ is an MN×PQ matrix, $g_{l}$ is a PQ×1 vector, and $e_{l}$ is the noise term. In (6):

(7) $$s_{l}={\left[s_{l}(t_{r,1},t_{a,1}),\cdots ,s_{l}(t_{r,N},t_{a,1}),s_{l}(t_{r,1},t_{a,2}),\cdots ,s_{l}(t_{r,N},t_{a,2}),\cdots ,s_{l}(t_{r,1},t_{a,M}),\cdots ,s_{l}(t_{r,N},t_{a,M})\right]}^{T}$$

Let:

(8) $$\begin{array}{l} a_{l}(t_{r,n},t_{a,m},i)=\exp\left[j\pi \gamma {\left(t_{r,n}-2R_{l}(t_{a,m},i)/c\right)}^{2}\right]\exp\left[-j4\pi f_{c}R_{l}(t_{a,m},i)/c\right];\\ \begin{array}{ll} a_{l,i}= & [a_{l}(t_{r,1},t_{a,1},i),\cdots ,a_{l}(t_{r,N},t_{a,1},i),a_{l}(t_{r,1},t_{a,2},i),\cdots ,\\ & a_{l}(t_{r,N},t_{a,2},i),\cdots ,a_{l}(t_{r,1},t_{a,M},i),\cdots ,a_{l}(t_{r,N},t_{a,M},i)] \end{array} \end{array}$$

then the projection matrix can be expressed as:

(9) $$A_{l}={\left[a_{l,1},a_{l,2},\cdots ,a_{l,i},\cdots ,a_{l,PQ}\right]}^{T}$$

Hence, the projection from the scene to the returned data of the *i*-th channel is obtained. Like Equation (6), the projections of other channels can also be obtained easily. Due to the same sampling area, the support sets of $g_{l}(l=1,2,\cdots ,L)$ are consistent, and the only difference is the backward scattering amplitude. In conclusion, the multichannel SAR imaging model can be expressed as:

(10) $$s=\left[\begin{array}{l} s_{1}\\ \vdots \\ s_{l}\\ \vdots \\ s_{L-1}\\ s_{L}\; \end{array}\right]=\mathrm{Ag}+e=\left[\begin{array}{l} A_{1}\\ \vdots \\ A_{l}\\ \vdots \\ A_{L-1}\\ A_{L}\; \end{array}\right]\left[\begin{array}{l} g_{1}\\ \vdots \\ g_{l}\\ \vdots \\ g_{L-1}\\ g_{L}\; \end{array}\right]+\left[\begin{array}{l} e_{1}\\ \vdots \\ e_{l}\\ \vdots \\ e_{L-1}\\ e_{L}\; \end{array}\right]$$

where s is an LMN×1 vector, corresponding to multichannel SAR returned signal; A is an LMN×PQ matrix, corresponding to the projection of the targets to the multichannel antennas; g is an LPQ×1 vector, corresponding to the backscattering amplitude of *L* channels; e is an LPQ×1 matrix, corresponding to the noise of each channel. Without loss of generality, the projection matrixs $A_{l}(l=1,2,\cdots ,L)$ is considered to be varied with different channels. In many situations, such as PolSAR and InSAR, the projection matrices $A_{l}(l=1,2,\cdots ,L)$ are the same in different channels.

**Figure 2:**
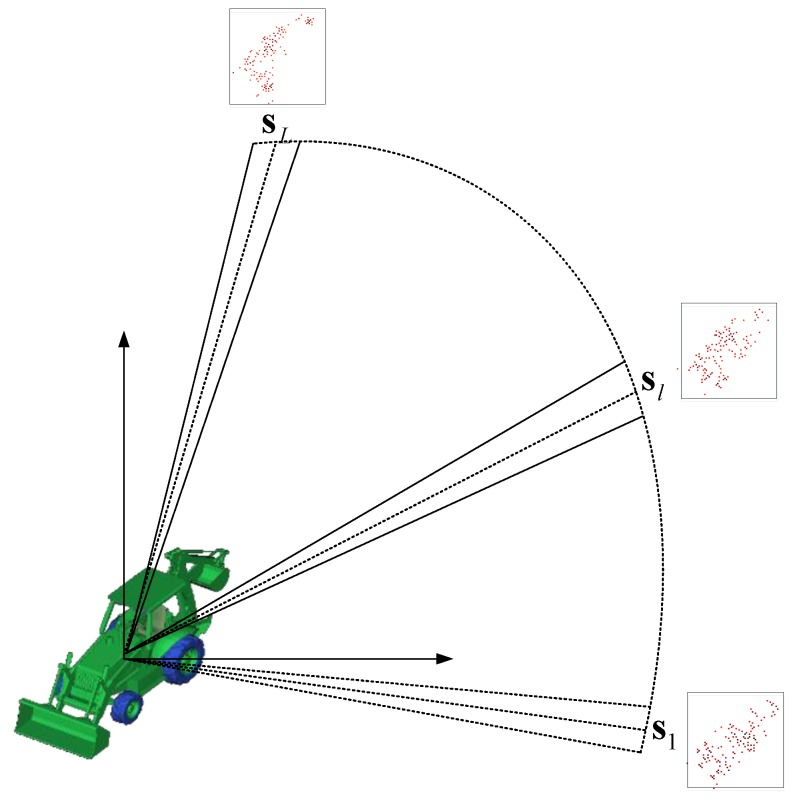
Wide-angle SAR data collection and reflectivity reconstruction geometry. The aircraft transmits pulses at the ground patch from a circular trajectory and reflectivity fields of the ground patch are reconstructed at a discrete set of aspects.

In wide-angle SAR imaging, due to the dependence of the reflectivity response on the aspect of an impinging electromagnetic wave, there exists a reflectivity map of a scene at each aspect. Assume that the imaging scene is interrogated and reconstructed at a number of different aspects. Denote the set of time observations at the *l*-th aspect as $s_{l}$ , denote the spatial reflectivity field at the *l*-th aspect as $g_{l}$ and denote the projection from the scene to the returned data of the *l*-th aspect as $A_{l}$ . Then the signal model of wide-angel SAR imaging can be also expressed the same as Equation (10). We emphasize the spatial geometry of the data collection, as well as aspect angles at which the spatial reflectivity fields are being reconstructed on Figure 2. This figure shows a target in the coordinate center and the aircraft’s circular trajectory at a large stand-off range, with the phase history returns over small sub-apertures tied to one spatial image.

## 3. Multichannel and Wide-Angle SAR Imaging Based on CS

### 3.1. The Scheme of Compressive Sampling

In order to apply a CS scheme to Equation (10), a reduced set of elements in s is selected randomly, and a reduced set of rows in A is also selected accordingly. Rigorously, this sampling process can be realized with a measurement matrix Φ . Let $s^{CS}=\Phi s$ be the down-sampled data selected from s randomly, $e^{CS}=\Phi e$ be the noise included with the signal $s^{CS}$ , $A^{CS}=\Phi A$ be the reduced projection matrix, the rows of which are selected from A accordingly. The CS measurement of the multichannel or wide-angle SAR signal can be expressed as:

(11) $$s^{CS}=\Phi s=\left[\begin{array}{cccc} \Phi_{1} & 0 & \cdots & 0\\ 0 & \Phi_{2} & \cdots & 0\\ \vdots & \vdots & \ddots & 0\\ 0 & 0 & \cdots & \Phi_{L} \end{array}\right]s=\Phi \mathrm{Ag}+\Phi e=A^{CS}g+e^{CS}$$

where $\Phi_{l}=\Phi_{l}^{a}⊗\Phi_{l}^{r}\left(l=1,2,\cdots ,L\right)$ represents the measurement matrix of each channel or subaperture, ⊗ represents the Kronecker product, $\Phi_{l}^{a}$ corresponds to the measurement submatrix in the azimuth direction, $\Phi_{l}^{r}$ corresponds to the measurement submatrix matrix in the range direction. In the radar system, we can realize the random range under-sampling by using a random A/D converter or random frequency synthesizer driven by set of random numbers [32]. For the azimuth under-sampling, there are two methods: random azimuth under-sampling and jittered azimuth under-sampling [12].

After compressive sampling, the sampling data $s^{CS}$ of the signal s is far from Nyquist sampling requirements, and thus the traditional SAR imaging methods cannot realize imaging efficiently. However, provided that the sparsity or compressibility condition holds, the targets can be reconstructed exactly by using the CS scheme. In radar imaging, many radar scenes are spatially sparse or can be sparsely represented [9,10,11], which meets the condition of CS application and thus CS can be applied into radar imaging.

### 3.2. Independent CS Processing Method

We can easily apply the conventional CS-based SAR imaging methods to each channel or sub-aperture. According to CS, g can be recovered by solving L optimization problems corresponding to each channel or sub-aperture:

(12) $$\min{\left\|g_{l}\right\|}_{0}\;\mathrm{suject}\;\mathrm{to}\;{\left\|s_{l}^{CS}-A_{l}^{CS}g_{l}\right\|}_{2}^{2}\leq \xi_{l}\;(l=1,2,\cdots L)$$

where ${\left\|\cdot \right\|}_{0}$ is defined as the number of the non-zero elements (also called sparsity or $l_{0}$ norm), min(·) denotes the minimization, $g_{l}$ is the backward scattering estimator of the *l*-th channel or sub-aperture with respect to $A_{l}^{CS}$ and $\xi_{l}$ is the noise level of the *l*-th channel or sub-aperture, which can be estimated by the range bins containing only noise [9]. When L optimization problems are successively solved, SAR images of all channels or sub-apertures are obtained accordingly.

Since Equation (12) is an NP-hard problem, namely computationally infeasible, in practice approximations of Equation (12) are explored to give approximate solutions. This optimization problem can be efficiently solved by both greedy algorithms such as orthogonal matching pursuit (OMP) [33] and regularized orthogonal matching pursuit (ROMP) [34] and compressive sampling matching pursuit (CoSaMP) [35], etc. An alternative solution uses $l_{1}$ norm instead of $l_{0}$ norm to convert it into a convex optimization problem [36], which is usually more precise but less efficient. To improve the computational efficiency of $l_{1}$ norm optimization problem, some algorithms are sequentially proposed, such as fast iterative shrinkage/thresholding algorithm (FISTA) [37], sparse reconstruction by separable approximation (SpaRSA) [38], and split augmented Lagrangian shrinkage algorithm (SALSA) [39]. SALSA is consistently and considerably faster than the state of the art methods FISTA and SpaRSA, due to the usage of the Hessian of the data fidelity term. In the case of SAR imaging, the signal for construction (i.e., the backward scattering coefficient vector after the discretization of the scene) is usually very long, so we need to choose some faster algorithms. Generally, the OMP algorithm is faster and easier to implement, and provides guarantees of exact recovery. In this paper, the OMP algorithm is selected to solve the optimization problem.

The purpose of the application of CS is to reduce the sampling data. The needed measurements is closely related to the sparsity *K*, namely the amount of dominant scatterers. According to the CS theory, the required number of target samples $N_{s}$ should satisfy $N_{s}=O(K\log(PQ/K))$ . In many scenes, the amount of dominant scatterers is usually much less than that of the pixels of the image plane. The more sparse nonzero pixels distribute over the image plane, the larger the data reduction rate can be set. In real applications, there always needs the statistical analysis of the imaging scene before the selection of the data reduction rate. For complex imaging scene, such as land, the nonzero pixels occupy most of the SAR image. In this case, the sparsity assumption cannot hold and thus the data reduction rate is very low. However, this does not mean that the CS theory cannot be applied in the complex scene. The scene can be sparsely represented by time-frequency transform, wavelet transform or adaptive sparse transform. We can recovery the coefficients of sparse transform with fewer measurements and then obtain the required SAR image by inverse sparse transform. The needed measurements is closely related to the number of nonzero coefficients. Therefore, it is very important to select suitable sparse transform for reducing the measurements.

After the estimation of the backward scattering coefficient vector g , we can obtain high- resolution SAR images of each channel or sub-aperture. Next, some valuable information can be extracted from the obtained images of each channel or sub-aperture. For example, InSAR exploits the phase difference of the SAR images from two closely separated antennas to obtain the height information of the target; PolSAR extracts polarization characteristic from the SAR images generated by each polarimetric channel data; wide-angel composite SAR image can be obtained by a nonlinear combination of these sub-aperture images, and the scattering shapes in the azimuth direction are also extracted, being helpful for target recognition. It should be noted that since the height, polarization characteristic or aspect scattering information is closely related with the phase or amplitude among all SAR images of different channels or sub-apertures, it is important to guarantee that the positions and number of scatterers in each channel or sub-aperture. However, independent CS processing cannot guarantee that the dominant scatterers are present in the same pixels in the different recovered high-resolution images. Besides, the independent channel CS processing cannot make full use of the increase of data rate to improve the SNR gain.

### 3.3. Joint CS Processing Method

In order to improve the performance of independent CS processing, in this subsection, a CS-based method for joint high-resolution imaging and information preservation is proposed. As each channel or sub-aperture observes the same area, the data in each channel or sub-aperture are strongly related. According the prior information, the multichannel or multi-aspect signals share the same sparsity support over all channels or sub-apertures, which means that the position of every scatterer is identical in all channel or sub-aperture images, but its amplitude may be different. In other words, the sparse solution has the nonzero elements at the same locations but different coefficients in all channels or sub-apertures. This makes sense, as the ensemble is jointly sparse. As a result, by concatenating the coefficient matrices $g_{l}$ of each channel or sub-aperture, a novel minimization sparsity-constraint term described the joint sparsity characteristic is formulated as:

(13) $${\left\|g\right\|}_{0}={\left\|\left|g_{1}\right|+\left|g_{2}\right|+\cdots +\left|g_{l}\right|+\cdots +\left|g_{L}\right|\right\|}_{0}$$

where ${\left\|g\right\|}_{0}$ denotes the mixed norm, which promotes the sparsity and captures the essence nature of signal ensemble. Then we can reconstruct multichannel or wide-angel SAR images simultaneously by solving the following minimization problem:

(14) $$\underset{g_{l}(l=1,2,\cdots ,L)}{\min}{\left\|g\right\|}_{0}\;\mathrm{suject}\;\mathrm{to}\;\left\{ \begin{array}{c} {\left\|s_{1}^{CS}-A_{1}^{CS}g_{1}\right\|}_{2}^{2}\leq \xi \\ {\left\|s_{2}^{CS}-A_{2}^{CS}g_{2}\right\|}_{2}^{2}\leq \xi \\ \vdots \\ {\left\|s_{L}^{CS}-A_{L}^{CS}g_{L}\right\|}_{2}^{2}\leq \xi \; \end{array}\right.$$

where ξ is chosen according to the channel with lowest noise level to make sure good SAR images can be obtained in each channel or sub-aperture.

Comparing Equations (12) with (14), the difference between the independent CS processing and joint CS processing methods lies in the sparsity-constraint term. Independent CS processing aims to minimize the sparsity-constraint term according to the sparse property of the signal in a single channel or sub-aperture, but it cannot guarantee the nonzero pixels present on the same positions in the different recovered high-resolution SAR images. In contrast, exploiting the joint sparsity characteristic of multichannel or multi-aspect signals, the sparsity-constraint term in the joint CS processing method minimizes most elements in the same locations from the measurements. As a consequence, the recovered SAR images in different channels or sub-apertures share the same number and locations of the scatterers and more valuable scattering information can be preserved during the imaging process.

To solve Equation (14), we propose an improved OMP algorithm to realize joint sparse recovery of multichannel or multi-aspect target vectors. The algorithm’s steps are as follows:

1. Initialize: Set the iteration counter *t* = 1. For each channel or sub-aperture index *l*, initialize the target vector $g_{l,0}=0$ , the support set $\Lambda_{0}=0$ , and the augmented matrix $\Phi_{l,0}=0$ , which will be composed of the selected vectors $A_{l}^{CS}$ according to the index set. Let $r_{l,t}^{CS}$ denote the residual of the signal $s_{l}$ remaining after the first *t* iterations, and initialize $r_{l,0}^{CS}=s_{l}^{CS}$ .
2. Find the index $\lambda_{t}$ that solves the easy optimization problem:
(15) $$\lambda_{t}=\underset{k=1,\cdots ,PQ}{\arg\max}\sum_{l=1}^{L}\left|\langle r_{l,t-1}^{CS},A_{l,k}^{CS}\rangle \right|$$
  where $A_{l,k}^{CS}$ is the *k*-th column vector of the matrix $A_{l}^{CS}$ .
3. Augment the support set and the matrix of chosen atoms: $\Lambda_{t}=\Lambda_{t-1}\cup \left\{\lambda_{t}\right\}$ and $\Phi_{l,t}=\left[\Phi \;A_{l,\lambda_{t}}^{CS}\right]$ .
4. Calculate the projection coefficient $g_{l,t}$ onto the span of the augmented matrix $\Phi_{l,t}$ , using standard techniques for least-squares problems:
(16) $$g_{l,t}=\underset{g_{l}}{\arg\min}{\left\|s_{l}^{CS}-\Phi_{l,t}g_{l}\right\|}_{2}\;(l=1,2,\cdots ,L)\;$$
5. Renew the residual signal $r_{l,t}^{CS}$ , i.e.:
(17) $$r_{l,t}^{CS}=s_{l}^{CS}-\Phi_{l,t}g_{l,t}\;(l=1,2,\cdots ,L)$$
6. Check for convergence: If ${\left\|r_{l,t}^{CS}\right\|}_{2}>\varepsilon {\left\|s_{l}^{CS}\right\|}_{2}$ for all l , then increment *t* and go to Step 2; otherwise, stop. The parameter ε determines the target error power level allowed for algorithm convergence.

Compared with the standard OMP algorithm when applied in the independent CS processing, the main difference of the proposed algorithm lies in Step 2. In the standard OMP algorithm, the sparsity support index set may be varied in different channels or sub-apertures because the index $\lambda_{t,l}$ is determined by the *l*th channel or sub-aperture itself:

(18) $$\lambda_{t,l}=\underset{k=1,\cdots ,PQ}{\arg\max}\left|\langle r_{l,t-1}^{CS},A_{l,k}^{CS}\rangle \right|\;(l=1,2,\cdots ,L)$$

As a result, the number or locations of the nonzero elements in the recovered target vectors may be also varied over different channels or sub-apertures. In contrast, the proposed algorithm selects the index $\lambda_{t}$ by processing the multichannel or multi-aspect signals jointly. Thus the support set is the same and the sparse solution has the nonzero elements on the same positions, namely the consistency of the number and the positions of scatterers in all channel or sub-aperture images. Therefore, the proposed method effectively overcomes the problem of nonalignment of scatterers in different channels or sub-apertures brought by the traditional independent CS processing.

The running time of the independent CS processing method using the standard OMP algorithm is dominated by the step of the index selection, whose total cost is $O(Lt_{end}N_{s}PQ)$ , where $N_{s}$ is the length of the sampled signal and $t_{end}$ is the iteration counter at the end of the algorithm [28]. The running time of the proposed method matches that of independent CS processing, only the increment of $Lt_{end}PQ$ addition. In practical operation, the increment of the computational complexity can almost be ignored.

## 4. Experimental Results and Analysis

### 4.1. Experimental Results for InSAR

**Figure 3:**
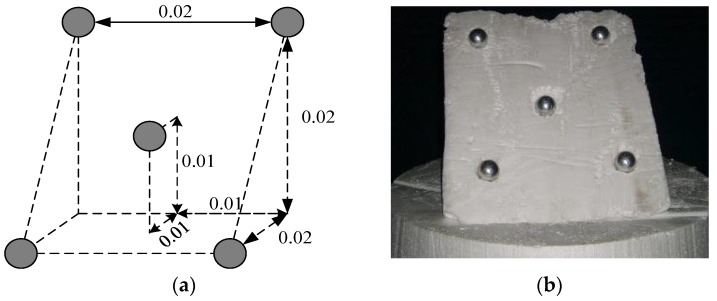
Target model of five metal balls. (**a**) 3-D spatial distribution; and (**b**) the photo in the anechoic chamber.

**Figure 4:**
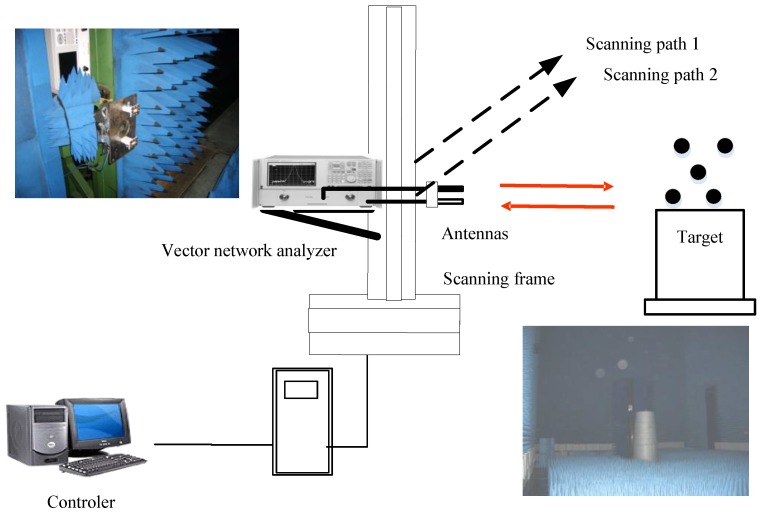
InSAR hardware-in-the-loop system constructed in the anechoic chamber.

The target model of five metal balls is shown in Figure 3. According to the anechoic chamber existing condition’s foundation, the InSAR hardware-in-the-loop system is constructed, as shown in Figure 3. The movement of the scanning frame along a slide guide simulates the flight trajectory of airborne SAR. Two closely consecutive scanning paths are used for the simulation of two closely separated antennas in the InSAR system. Figure 4 shows the target model of five metal balls. The test parameters are listed as follows: the vertical distance between the scanning path and the target is 2 m, the center frequency is 10 GHz, the baseline length between two scanning paths is 0.02 m; the system bandwidth is 4 GHz, the step size of the frequency is 40 MHz, the width of the scanning path is 1 m and there are 51 sampling points.

**Figure 5:**
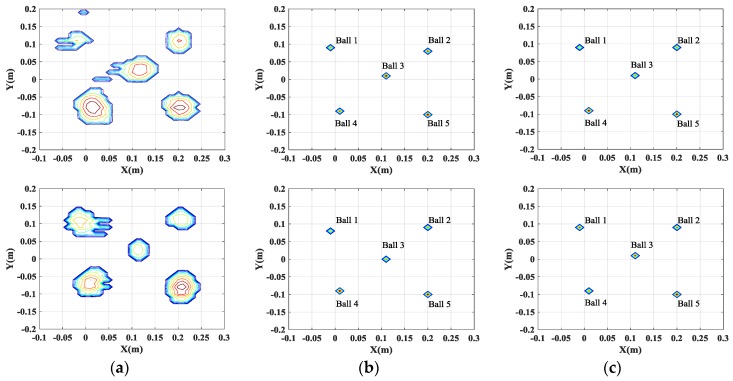
Comparison of imaging results by different imaging methods. Images in the first row are from the results of channel 1. Images in the second row are from the results of channel 2. (**a**) FBP method; (**b**) single channel CS method; and (**c**) multichannel joint CS method.

**Figure 6:**
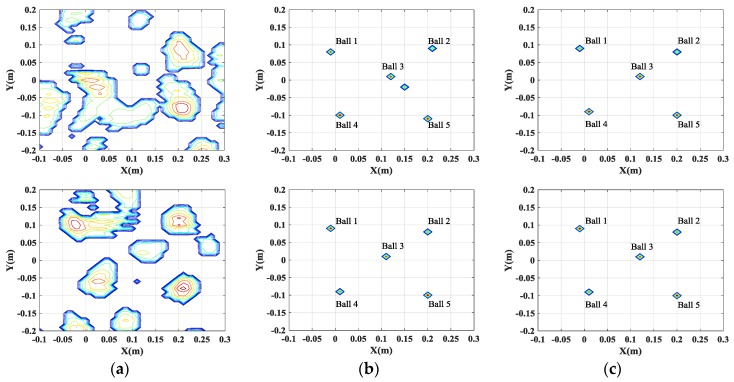
Comparison of imaging results by different imaging methods using 25% of full data. Images in the first row are from the results of channel 1. Images in the second row are from the results of channel 2. (**a**) FBP method; (**b**) single channel CS method; and (**c**) multichannel joint CS method.

Figure 5 and Figure 6 show comparisons of the imaging results obtained by different methods, when the full and partial data is used, respectively. It is apparent that the resolution of 2-D SAR images obtained by the conventional filtered back-projection (FBP) method is low because of high sidelobes, leading to difficulties in target identification. Moreover, the FBP method cannot reconstruct high-quality SAR images when 25% of the full data is used. On the contrary, the CS-based methods (including single channel CS and multichannel joint CS methods) can effectively restrain the sidelobes and mitigate the blurring effect to obtain the well-focused images. Especially, when 25% of the full data is used, the CS-based methods can still guarantee the accuracy of the reconstruction.

It is widely known that, for InSAR, the height information of the target can be estimated from the phase difference between the SAR images generated by different channels. Thus, it is important to preserve the cross-channels information. As shown in Table 1 and Table 2, the single channel CS independent processing cannot guarantee that the dominant scatterers are present in the same pixels in the different generated SAR images.

**Figure 7:**
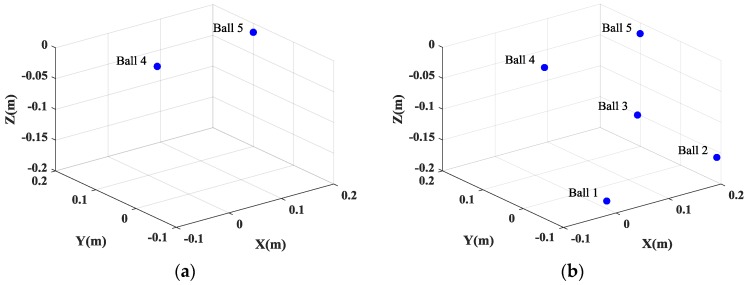
The recovered height information from the generated images. (**a**) Single channel CS method; and (**b**) multichannel joint CS method.

**Figure 8:**
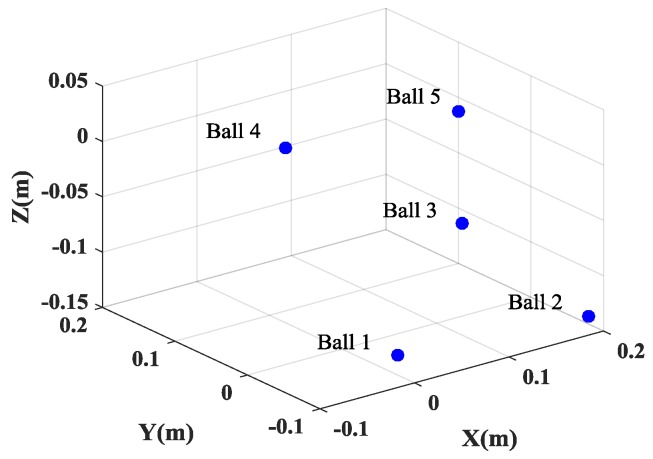
The height estimation from the generated SAR images by the multichannel joint CS method when 25% of full data is used.

This inconsistency of the positions of the scatterers in different SAR images will lead to the failure of the height estimation of the target. Figure 7a gives the height estimation of the target when the full data is used. It is shown that the height information of only two balls can be obtained. And even worse, when 25% of the full data is used, the positions of five balls are not consistent, which will lead to the entire failure of the height estimation of the target. However, by exploiting the joint sparsity property of the multichannel signals, multichannel joint CS method can guarantee the consistency of the positions of all scatterers in different channels and preserve the cross-channels information, which is beneficial for the height estimation of scatterers, as shown in Figure 7b and Figure 8. Besides, multichannel joint CS processing need fewer samples for reconstruction than single channel CS processing, due to the usage of the complementation of multichannel signals.

### 4.2. Experimental Results for PolSAR

#### 4.2.1. Electromagnetic Simulation Data of Backhoe

**Figure 9:**
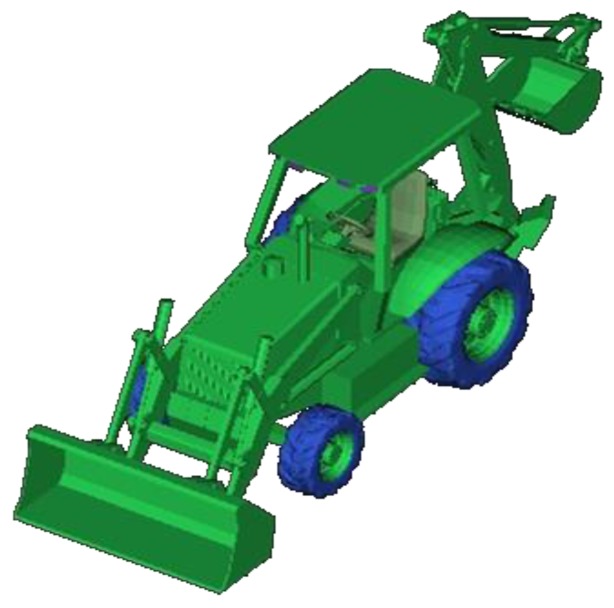
Three-dimensional CAD model of the backhoe.

To verify the effectiveness of the proposed method when applied for PolSAR, we firstly use the backhoe data which is provided in the website of https://www.sdms.afrl.af.mil [40]. The data consists of simulated wideband (7–13 GHz), full polarization, complex backscatter data from a backhoe vehicle in free space. A three-dimensional CAD model of the backhoe is shown in Figure 9. In this experiment, we only select full polarization data with the center frequency of 10 GHz, the bandwidth of 500 MHz and the angular aperture is 5°.

**Figure 10:**
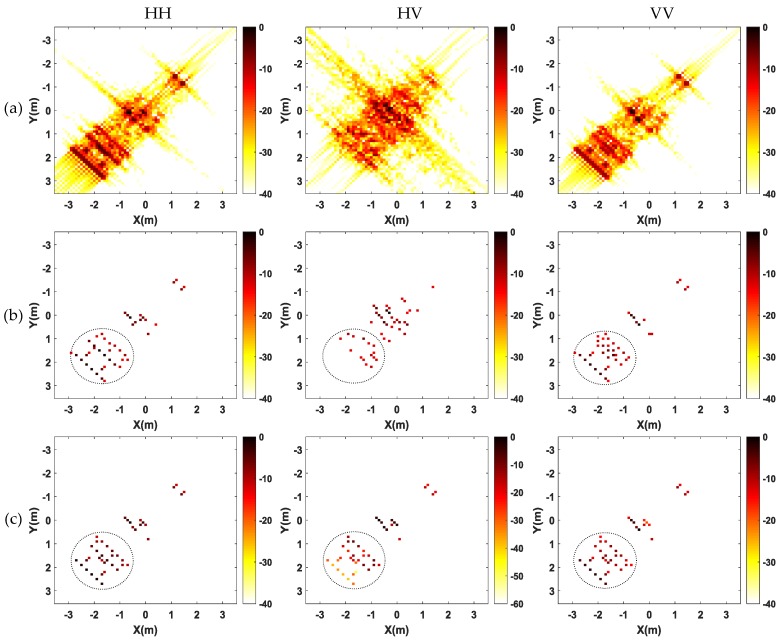
Comparison of imaging results by different imaging methods. (**a**) Traditional filtered back-projection method; (**b**) single channel CS method; and (**c**) multichannel joint CS method.

Figure 10 shows the comparison of full polarization SAR imaging results obtained by different methods. It is apparent polarization that full polarization SAR images generated by conventional FBP method are of low resolution and have high sidelobes, leading to difficulty in target identification. However, results generated by CS methods (both single channel CS and multichannel joint CS methods) show higher resolution and much less sidelobes. As shown in Figure 10b, the single channel CS independent processing cannot guarantee that the dominant scatterers are present in the same pixels among the generated SAR images from different channels, leading to deteriorate the cross-channels information. In contrast, by exploiting the joint sparsity property of the multichannel signals, the multichannel joint CS method can realize joint high-resolution imaging and cross-channels information preservation, effectively improving the recovery accuracy of scatterers, as shown in Figure 10c.

**Figure 11:**
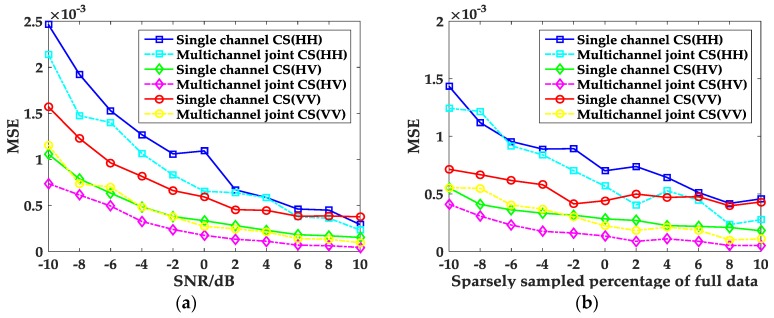
Relationship between the MSE of amplitude for each channel and SNRs and sparse samples. (**a**) SNRs; and (**b**) sparsely sampling percentage of full data.

Next, the performance of the proposed method at varying noise levels and with different sparse samples will be discussed. In order to show the performance of the proposed method in the presence of noise, SNRs ranging from −10 to 10 dB are tested with 50 trials carried out at each SNR level. In a similar way, different sparsely sampled data are tested with 50 trials for each sampled data, so as to test the performance of the proposed method when sparsely sampled data are used. For each SNR and sampled data, the mean square error (MSE) of the amplitude for each channel is calculated and depicted in Figure 11. It can be observed in Figure 11 that when the SNR and the sampling ratio increases, the MSE of the amplitude for each channel quickly decreases; multichannel joint CS method can reconstruct full polarization SAR images with fewer measurements and have stronger suppression capability than the single channel CS method.

#### 4.2.2. Real Measured Data in the Anechoic Chamber

**Figure 12:**
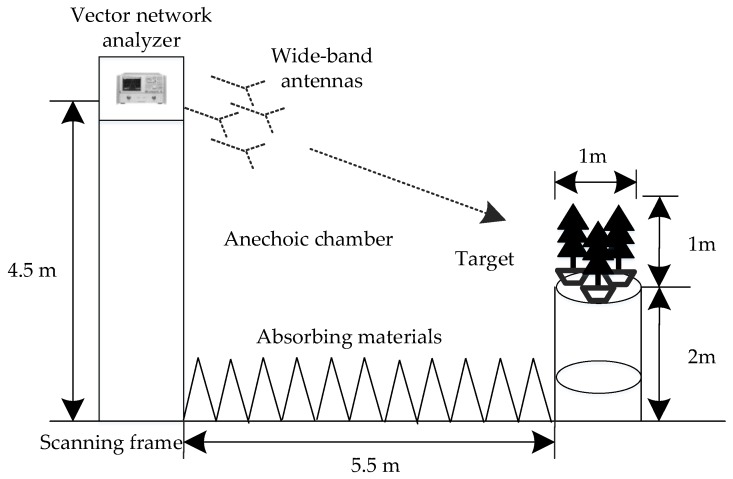
The measurement system of PolSAR.

**Figure 13:**
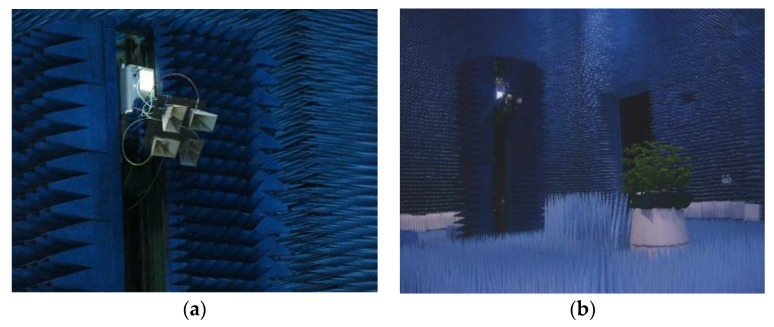
Photos of the PolSAR system in the anechoic chamber. (**a**) Antenna layout; and (**b**) the whole scene.

Experimental results are presented with the usage of real data acquired in the anechoic chamber to demonstrate the validity of the proposed method. As shown in Figure 12, the PolSAR hardware-in-the-loop system consists of a scanning frame, a vector network analyzer, four wideband antennas, and the scene including three trees. The movement of the scanning frame along a slide guide simulates the flight trajectory of airborne SAR. During the data acquisition, four antennas are employed with two transmitting horizontal (H) and vertical (V) polarization waves, and the other two receiving H and V polarization returns, which is shown in Figure 13a. It should be noted that due to the hardware limitation, the polarimetric measurement is not simultaneous, which means that the polarization waveform is alternately transmitted between H and V polarization, that is, first H polarization, then V polarization, etc. When the H polarization waveform is transmitted, the echoes of HH and HV polarization channels can be obtained simultaneously. When the V polarization waveform is transmitted, the echoes of VH and VV polarization channels can be obtained simultaneously. The whole observed scene in the anechoic chamber is shown in Figure 13b. Significant system parameters are given in Table 3.

**Figure 14:**
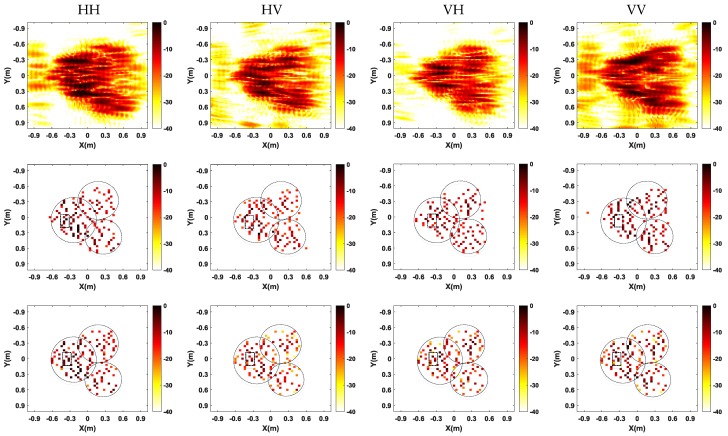
Comparison of imaging results by different imaging methods. Up: traditional filtered back-projection method; Middle: single channel CS method; and down: multichannel joint CS method.

Figure 14 shows the comparison of imaging results in four polarization channels obtained by different methods. It is apparent in Figure 14 that the SAR images obtained by conventional FBP method are of low resolution and have high sidelobes, which is not rich enough for further SAR image interpretation. SAR images generated by CS-based imaging methods (both single channel CS and multichannel joint CS methods) show a much higher resolution with much less sidelobes. By observing the SAR images in the second row in Figure 14 generated by single channel CS method, the number and positions of scatterers are not aligned in different channels. As a result, it is not easy to acquire the corresponding polarimetric scattering matrix of each scatterer and polarization information of some scatterers may be lost. While in results generated by multichannel joint CS method depicted in the third row in Figure 14, the number and positions of all scatterers in all channels are consistent; thus, the corresponding polarization information of each scatterer can be obtained. In order to show the advantage of multichannel joint CS method, we select some the strongest scatterers in the region marked by the dash squares in the second and third rows in Figure 14. The coordinates of the marked scatterers in the second row are changing in different channels, and some scatterers existed in one channel cannot be constructed in other channels. While in the third row, all scatterers are aligned, and only their amplitudes are different. Furthermore, the approximate shape of three trees is marked by the dash circles in the second and third rows in Figure 14. Compared with the SAR images in the second row, the SAR images in the third row are more close to the real scene, which is more beneficial for target recognition.

**Figure 15:**
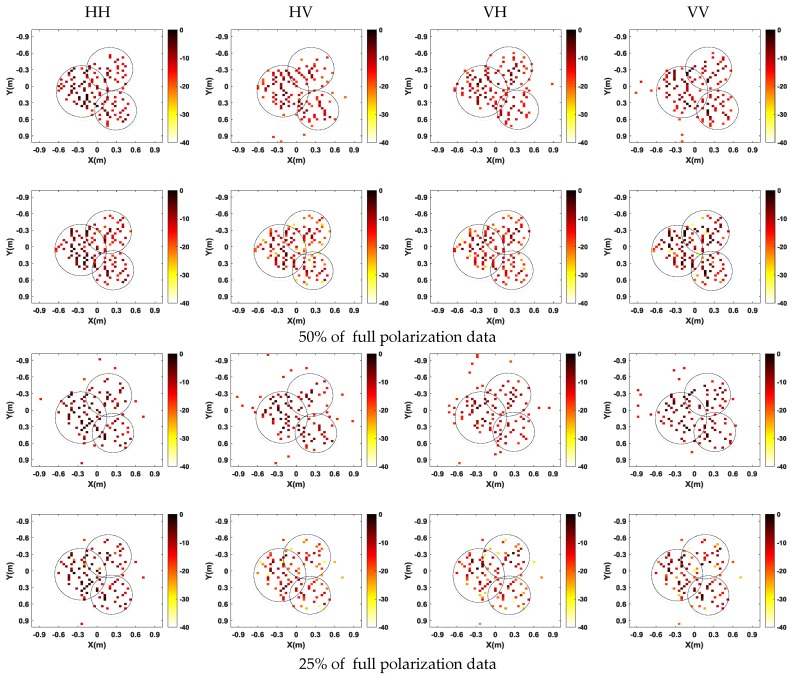
Imaging results for 50% and 30% of full polarization data. Up: single channel CS method; and down: multichannel joint CS method.

Next, in the CS framework, we randomly select samples with sparse frequencies and scanning locations. Figure 15 shows the comparison of imaging results from 50% and 25% of the full data by two CS-based methods. As is shown in Figure 15, compared with the single channel CS method, the imaging performance of multichannel joint CS method has a smaller influence when the sampled data decreases. It means that multichannel joint CS method can obtain focused SAR images with fewer measurements, which will be beneficial for further reducing sampled data of the SAR system. Besides, multichannel joint CS method still preserves the cross-channels information, guaranteeing the consistency of the number and positions of scatterers.

### 4.3. Experimental Results for Wide-Angle SAR

**Figure 16:**
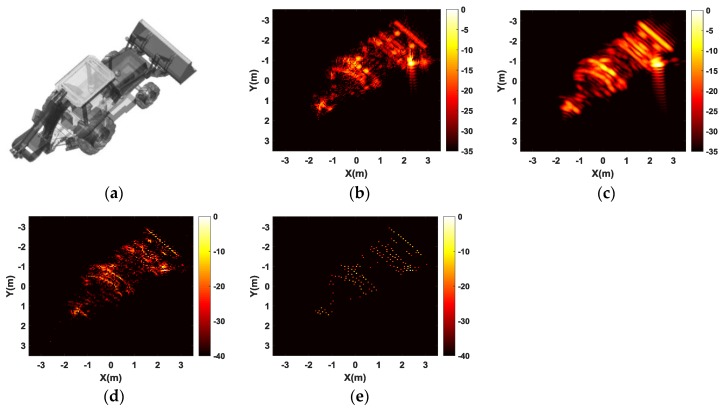
Comparison of imaging results by different imaging methods. (**a**) The backhoe CAD model; (**b**) FBP method applied on the full aperture of 65°; and composite images of (**c**) FBP method; (**d**) independent CS reconstruction and (**e**) joint CS reconstruction of 22 images each corresponding to a sub-aperture of 5°.

To verify the effectiveness of the proposed method when applied for wide-angle SAR composite image formation, we also use the backhoe data which is provided in the website of https://www.sdms.afrl.af.mil [40]. The CAD model of the backhoe is given in Figure 16a. In this experiment, we only select VV polarization data with the frequency bandwidth of 1 GHz around the center frequency of 10 GHz, the whole angular aperture of 65°. The reconstruction grid is chosen such that one 121 × 121 spatial image is reconstructed every 5°. Thus, there are total of 25 jointly reconstructed images corresponding to 25 consecutive, overlapping viewing aspects. Note that these images are plotted in dB scale, by first thresholding small values to zero at the same threshold level for both joint and independent CS reconstructions. The composite image results show the backhoe’s reflectivity in much finer detail when compared to results of FBP method applied to the full aperture data. The composite image by traditional FBP method is of low resolution and has high sidelobes due to only small sub-aperture. However, the composite image by the CS-based method, including independent and joint CS processing methods, show a much higher resolution with much less sidelobes. Besides, spatial support of the composite image by joint CS processing method is much smaller and only the dominant features are reconstructed. Although independent CS reconstructions also identify dominant features, some false scatterers appear in the reconstructed image.

**Figure 17:**
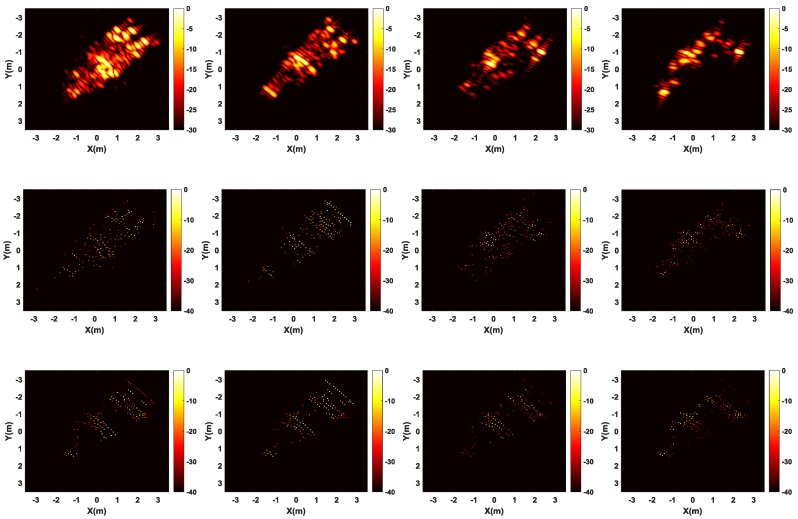
Three method’s reconstructed SAR images each of 5° width with maximum number of measurements. Columns left to right correspond to images centered at −7.5°, 2.5°, 15°, 27.5° degrees azimuth. Rows correspond to FBP method (top row), independent CS processing (middle row) and joint CS processing (bottom row).

Figure 17 shows the magnitudes of the backhoe’s spatial reflectivity when viewed from several reconstruction angles. Independent and joint CS processing methods produce better focused imagery, whereas the images reconstructed by the FBP method have noticeable sidelobes which is not beneficial for target identification. Independent CS reconstruction yields more magnitude responses, while joint CS reconstruction produces images with more compact spatial support.

**Figure 18:**
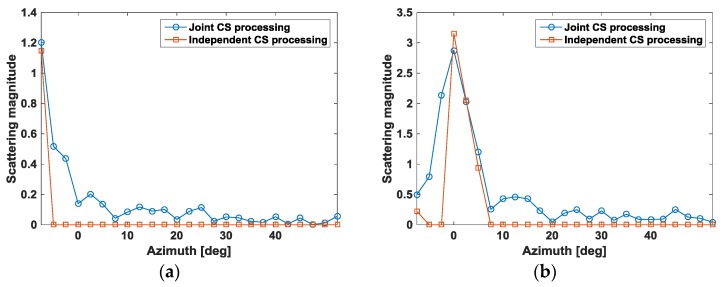

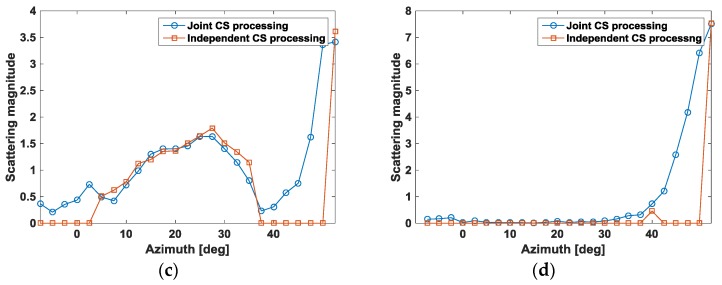
Magnitude of reflectivity response over full range of aspects for several sample pixels. (**a**) Sample pixel 1; (**b**) Sample pixel 2; (**c**) Sample pixel 3 and (**d**) sample pixel 4.

In Figure 18 we present reconstructed scattering shapes as a function of azimuth for a set of sample pixels. As expected, the target aspect scattering behavior has limited persistence. The fine detail provided in these plots allows for a scatterter feature extraction. For example, scatterers such as flat, metal plates have glint anisotropy that is very thin in azimuth, whereas flag and metal poles act as isotropic point scatters. Note that joint CS processing typically produces smooth scattering shapes, whereas independent CS processing reconstructs shapes that are jittery.

## 5. Conclusions

Based on CS theory, a novel multichannel and wide-angle SAR joint high-resolution imaging and information preservation method is proposed in this paper. By using the joint sparsity of the signal ensemble, compressive sampling in the range and azimuth directions, and improved OMP algorithm to solve the joint sparse recovery problem, the proposed method can use fewer measurements to realize high-resolution imaging and preserve the cross-channels or cross-sub-apertures information during the image formation, which is beneficial for the extraction of some valuable scattering information and reducing the data storage cost. Finally, experimental data processing results are used for verifying the validity of the proposed method.

Although a point-like target model is applied, which is reasonable according to the experimental data results, it may be an issue in imaging a complex target with extended scatterers. In these cases, the scattering response cannot be well described by the point-like target model, and thus, the sparsity assumption may not hold. Future work will focus on SAR imaging in a more complex scene.

## Figures and Tables

**Table 1:** The reconstructed positions of each metal ball by different CS-based methods using full data.

Method	Channel	Ball 1	Ball 2	Ball 3	Ball 4	Ball 5
Single channel CS method	Channel 1	**(−0.01, 0.09)**	**(0.20, 0.08)**	**(0.11, 0.01)**	(0.01, −0.09)	(0.20, −0.1)
	Channel 2	**(−0.01, 0.08)**	**(0.20, 0.09)**	**(0.11, 0.00)**	(0.01, −0.09)	(0.20, −0.1)
Multichannel joint CS method	Channel 1	(−0.01, 0.09)	(0.20, 0.09)	(0.11, 0.01)	(0.01, −0.09)	(0.20, −0.1)
	Channel 2	(−0.01, 0.09)	(0.20, 0.09)	(0.11, 0.01)	(0.01, −0.09)	(0.20, −0.1)

**Table 2:** The reconstructed positions of each metal ball when 25% of full data is used.

Method	Channel	Ball 1	Ball 2	Ball 3	Ball 4	Ball 5
Single channel CS method	Channel 1	**(−0.01, 0.08)**	**(0.21, 0.09)**	**(0.12, 0.01)**	**(0.01, −0.10)**	**(0.20, −0.11)**
	Channel 2	**(−0.01, 0.09)**	**(0.20, 0.08)**	**(0.11, 0.01)**	**(0.01, −0.09)**	**(0.20, −0.10)**
Multichannel joint CS method	Channel 1	(−0.01, 0.09)	(0.20, 0.08)	(0.12, 0.01)	(0.01, −0.09)	(0.20, −0.1)
	Channel 2	(−0.01, 0.09)	(0.20, 0.08)	(0.12, 0.01)	(0.01, −0.09)	(0.20, −0.1)

**Table 3:** System parameters.

Parameter	Value
Carrier Frequency	10 GHz
System Bandwidth	1 GHz
Frequency Step	12.5 MHz
Number of Transmitted Frequencies	81
Scanning Length	3 m
Scanning Interval	15 mm
Measuring distance	5 m
Look-down angle	20°
